# 3-(3,4-Dichloro­phen­yl)-1-(2-naphth­yl)prop-2-en-1-one

**DOI:** 10.1107/S1600536809034084

**Published:** 2009-08-29

**Authors:** Zhi-Ke Lu, Yan Feng, Shen Li, Hai-Lin Diao

**Affiliations:** a Forestry College, Guangxi University, Nanning 530005, People’s Republic of China; b Chemistry Department, Guangxi Industrial Vocational Technical College, Nanning 530001, People’s Republic of China

## Abstract

The asymmetric unit of the title compound, C_19_H_12_Cl_2_O, contains four independent mol­ecules, which can be divided into two pairs of mol­ecules with close values of the C—C(=O)—C=C torsion angles in each pair, *viz.* 165.12 (16) and 165.68 (15)° in one pair, and −164.66 (15) and −164.81 (15)° in the other pair. The crystal packing exhibits short inter­molecular Cl⋯Cl contacts of 3.362 (1) Å.

## Related literature

For a related structure, see Lu *et al.* (2006 ▶).
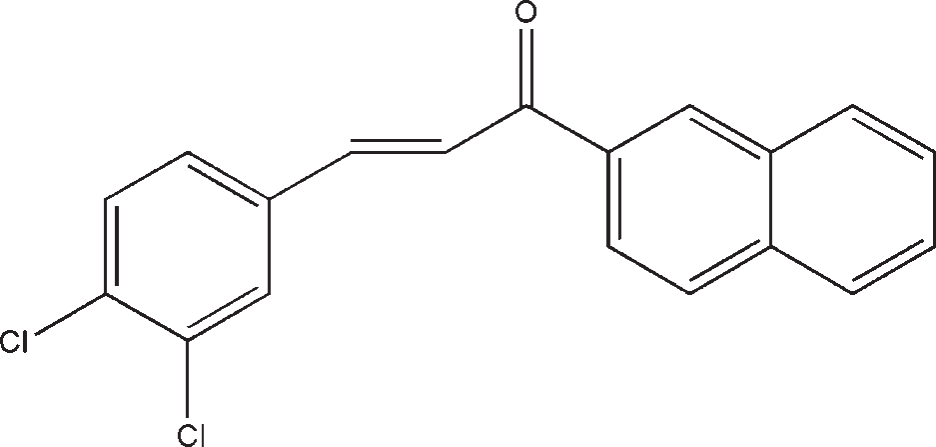


## Experimental

### 

#### Crystal data

C_19_H_12_Cl_2_O
*M* *_r_* = 327.19
Triclinic, 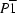

*a* = 7.5273 (10) Å
*b* = 11.5212 (14) Å
*c* = 33.301 (4) Å
α = 92.338 (7)°
β = 96.469 (7)°
γ = 90.125 (6)°
*V* = 2867.1 (6) Å^3^
*Z* = 8
Mo *K*α radiation
μ = 0.45 mm^−1^
*T* = 113 K
0.36 × 0.34 × 0.28 mm

#### Data collection

Rigaku Saturn CCD area-detector diffractometer
Absorption correction: multi-scan (*CrystalClear*; Rigaku, 2005 ▶) *T*_min_ = 0.855, *T*_max_ = 0.884
26590 measured reflections
13067 independent reflections
10135 reflections with *I* > 2σ(*I*)
*R*_int_ = 0.023

#### Refinement

*R*[*F* ^2^ > 2σ(*F* ^2^)] = 0.036
*wR*(*F* ^2^) = 0.106
*S* = 1.07
13067 reflections
793 parameters
H-atom parameters constrained
Δρ_max_ = 0.37 e Å^−3^
Δρ_min_ = −0.44 e Å^−3^

### 

Data collection: *CrystalClear* (Rigaku, 2005 ▶); cell refinement: *CrystalClear*; data reduction: *CrystalClear*; program(s) used to solve structure: *SHELXS97* (Sheldrick, 2008 ▶); program(s) used to refine structure: *SHELXL97* (Sheldrick, 2008 ▶); molecular graphics: *SHELXTL* (Sheldrick, 2008 ▶); software used to prepare material for publication: *CrystalStructure* (Rigaku/MSC, 2004 ▶).

## Supplementary Material

Crystal structure: contains datablocks global, I. DOI: 10.1107/S1600536809034084/cv2599sup1.cif

Structure factors: contains datablocks I. DOI: 10.1107/S1600536809034084/cv2599Isup2.hkl

Additional supplementary materials:  crystallographic information; 3D view; checkCIF report

## Figures and Tables

**Fig. 1.**
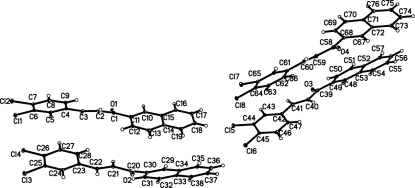
Four independent molecules of (I), shown with 30% probability displacement ellipsoids (arbitrary spheres for H atoms).

## supplementary crystallographic information

### Comment

The title compound, (I), (Fig. 1) was prepared and structurally characterized as a part of our ongoing studies of pyrazoline derivatives (Lu *et al.*, 2006).

### Experimental

A mixture of 3,4-dichlorobenzaldehyde(5 mmol) and 2-(prop-1-en-2-yl) naphthalene benzaldehyde (10 mmol) was dissolved in aqueous ethanol (100 ml, 60% *v*/*v*). The solution of KOH (4.0 ml, 7.1 mmol) was slowly added and stirred at 323 K for 5 h. The resulting mixture was neutralized with 2 N HCl. The precipitate filtered off, washed with water, and crystallized from dichloromethane-methanol (1/4 *v*/*v*) to obtain (I) (m.p.426–427k). Crystals suitable for X-ray analysis were grown by slow evaporation of a methanol-dichloromethane (4/1 *v*/*v*) solution at room temperature over a period of 16 d.

### Refinement

All H atoms were placed geometrically (C—H = 0.95 Å), and refined as riding with *U*_iso_(H) = 1.2*U*_eq_(C).

### Crystal data

**Table:**

C_19_H_12_Cl_2_O	*Z* = 8
*M**_r_* = 327.19	*F*(000) = 1344
Triclinic, *P*1	*D*_x_ = 1.516 Mg m^−^^3^
Hall symbol: -P 1	Melting point: 426-427 K K
*a* = 7.5273 (10) Å	Mo *K*α radiation, λ = 0.71070 Å
*b* = 11.5212 (14) Å	Cell parameters from 4591 reflections
*c* = 33.301 (4) Å	θ = 1.2–27.2°
α = 92.338 (7)°	µ = 0.45 mm^−^^1^
β = 96.469 (7)°	*T* = 113 K
γ = 90.125 (6)°	Block, yellow
*V* = 2867.1 (6) Å^3^	0.36 × 0.34 × 0.28 mm

### Data collection

**Table:**

Rigaku Saturn CCD area-detector diffractometer	13067 independent reflections
Radiation source: Rotating anode	10135 reflections with *I* > 2σ(*I*)
confocal	*R*_int_ = 0.023
Detector resolution: 7.31 pixels mm^-1^	θ_max_ = 27.9°, θ_min_ = 1.9°
ω and φ scans	*h* = −9→9
Absorption correction: multi-scan (*CrystalClear*; Rigaku, 2005)	*k* = −15→14
*T*_min_ = 0.855, *T*_max_ = 0.884	*l* = −43→43
26590 measured reflections	

### Refinement

**Table:**

Refinement on *F*^2^	Primary atom site location: structure-invariant direct methods
Least-squares matrix: full	Secondary atom site location: difference Fourier map
*R*[*F*^2^ > 2σ(*F*^2^)] = 0.036	Hydrogen site location: inferred from neighbouring sites
*wR*(*F*^2^) = 0.106	H-atom parameters constrained
*S* = 1.07	*w* = 1/[σ^2^(*F*_o_^2^) + (0.0577*P*)^2^ + 0.5613*P*] where *P* = (*F*_o_^2^ + 2*F*_c_^2^)/3
13067 reflections	(Δ/σ)_max_ = 0.001
793 parameters	Δρ_max_ = 0.37 e Å^−^^3^
0 restraints	Δρ_min_ = −0.44 e Å^−^^3^

### Special details

**Table:**

Geometry. All e.s.d.'s (except the e.s.d. in the dihedral angle between two l.s. planes) are estimated using the full covariance matrix. The cell e.s.d.'s are taken into account individually in the estimation of e.s.d.'s in distances, angles and torsion angles; correlations between e.s.d.'s in cell parameters are only used when they are defined by crystal symmetry. An approximate (isotropic) treatment of cell e.s.d.'s is used for estimating e.s.d.'s involving l.s. planes.
Refinement. Refinement of *F*^2^ against ALL reflections. The weighted *R*-factor *wR* and goodness of fit *S* are based on *F*^2^, conventional *R*-factors *R* are based on *F*, with *F* set to zero for negative *F*^2^. The threshold expression of *F*^2^ > σ(*F*^2^) is used only for calculating *R*-factors(gt) *etc*. and is not relevant to the choice of reflections for refinement. *R*-factors based on *F*^2^ are statistically about twice as large as those based on *F*, and *R*-factors based on ALL data will be even larger.

### Fractional atomic coordinates and isotropic or equivalent isotropic displacement parameters (Å^2^)

**Table:**

	*x*	*y*	*z*	*U*_iso_*/*U*_eq_	
Cl1	−0.00530 (6)	0.47380 (4)	−0.209773 (11)	0.02137 (10)	
Cl2	−0.18280 (6)	0.22714 (4)	−0.206473 (12)	0.02169 (10)	
Cl3	0.48364 (6)	−0.02522 (3)	−0.207594 (11)	0.02037 (9)	
Cl4	0.32062 (6)	0.22617 (3)	−0.204037 (11)	0.02038 (9)	
Cl5	0.80421 (6)	0.47713 (3)	0.292686 (11)	0.02060 (9)	
Cl6	0.97202 (6)	0.22757 (4)	0.295246 (12)	0.02159 (10)	
Cl7	0.29865 (6)	−0.02818 (3)	0.291430 (11)	0.02085 (9)	
Cl8	0.47357 (6)	0.22092 (3)	0.293851 (12)	0.02095 (9)	
O1	0.27954 (17)	0.60599 (10)	0.01527 (3)	0.0239 (3)	
O2	0.78508 (17)	−0.10351 (10)	0.01670 (3)	0.0233 (3)	
O3	0.73173 (17)	0.60659 (10)	0.51678 (3)	0.0235 (3)	
O4	0.23484 (17)	−0.10235 (10)	0.51615 (3)	0.0234 (3)	
C1	0.2845 (2)	0.50007 (14)	0.01544 (5)	0.0163 (3)	
C2	0.2051 (2)	0.42617 (14)	−0.01949 (5)	0.0189 (3)	
H2	0.1829	0.3461	−0.0161	0.023*	
C3	0.1646 (2)	0.47136 (14)	−0.05569 (5)	0.0165 (3)	
H3	0.1938	0.5509	−0.0580	0.020*	
C4	0.0788 (2)	0.40906 (13)	−0.09209 (4)	0.0152 (3)	
C5	0.0749 (2)	0.46242 (14)	−0.12922 (5)	0.0159 (3)	
H5	0.1267	0.5374	−0.1303	0.019*	
C6	−0.0041 (2)	0.40647 (13)	−0.16443 (4)	0.0153 (3)	
C7	−0.0807 (2)	0.29715 (13)	−0.16304 (4)	0.0156 (3)	
C8	−0.0789 (2)	0.24368 (14)	−0.12628 (5)	0.0168 (3)	
H8	−0.1328	0.1694	−0.1253	0.020*	
C9	0.0016 (2)	0.29893 (13)	−0.09119 (5)	0.0165 (3)	
H9	0.0045	0.2616	−0.0662	0.020*	
C10	0.3765 (2)	0.49862 (13)	0.08884 (5)	0.0147 (3)	
H10	0.3281	0.5745	0.0907	0.018*	
C11	0.3692 (2)	0.44034 (13)	0.05170 (4)	0.0153 (3)	
C12	0.4422 (2)	0.32751 (14)	0.04866 (5)	0.0181 (3)	
H12	0.4337	0.2865	0.0232	0.022*	
C13	0.5251 (2)	0.27731 (14)	0.08231 (5)	0.0177 (3)	
H13	0.5781	0.2030	0.0797	0.021*	
C14	0.5329 (2)	0.33478 (13)	0.12094 (5)	0.0150 (3)	
C15	0.4553 (2)	0.44688 (13)	0.12426 (5)	0.0148 (3)	
C16	0.4588 (2)	0.50344 (14)	0.16303 (5)	0.0174 (3)	
H16	0.4064	0.5779	0.1656	0.021*	
C17	0.5371 (2)	0.45125 (15)	0.19667 (5)	0.0204 (3)	
H17	0.5388	0.4898	0.2225	0.024*	
C18	0.6154 (2)	0.34067 (15)	0.19333 (5)	0.0214 (4)	
H18	0.6694	0.3054	0.2169	0.026*	
C19	0.6144 (2)	0.28379 (14)	0.15648 (5)	0.0187 (3)	
H19	0.6685	0.2096	0.1546	0.022*	
C20	0.7887 (2)	0.00248 (14)	0.01717 (5)	0.0171 (3)	
C21	0.7086 (2)	0.06819 (14)	−0.01763 (5)	0.0189 (3)	
H21	0.6868	0.1490	−0.0142	0.023*	
C22	0.6668 (2)	0.01464 (14)	−0.05379 (5)	0.0165 (3)	
H22	0.6953	−0.0654	−0.0562	0.020*	
C23	0.5808 (2)	0.06863 (13)	−0.09011 (4)	0.0149 (3)	
C24	0.5721 (2)	0.00596 (13)	−0.12721 (5)	0.0151 (3)	
H24	0.6211	−0.0699	−0.1284	0.018*	
C25	0.4922 (2)	0.05398 (13)	−0.16234 (4)	0.0153 (3)	
C26	0.4207 (2)	0.16467 (13)	−0.16086 (4)	0.0154 (3)	
C27	0.4271 (2)	0.22752 (14)	−0.12403 (5)	0.0167 (3)	
H27	0.3765	0.3029	−0.1230	0.020*	
C28	0.5071 (2)	0.18000 (14)	−0.08905 (5)	0.0167 (3)	
H28	0.5120	0.2234	−0.0641	0.020*	
C29	0.8808 (2)	0.02162 (13)	0.09061 (5)	0.0149 (3)	
H29	0.8327	−0.0540	0.0925	0.018*	
C30	0.8731 (2)	0.07115 (13)	0.05331 (4)	0.0154 (3)	
C31	0.9453 (2)	0.18380 (14)	0.05041 (5)	0.0180 (3)	
H31	0.9365	0.2188	0.0249	0.022*	
C32	1.0276 (2)	0.24257 (14)	0.08396 (5)	0.0172 (3)	
H32	1.0799	0.3166	0.0814	0.021*	
C33	1.0355 (2)	0.19405 (13)	0.12265 (5)	0.0155 (3)	
C34	0.9595 (2)	0.08219 (13)	0.12592 (4)	0.0149 (3)	
C35	0.9641 (2)	0.03472 (14)	0.16475 (5)	0.0174 (3)	
H35	0.9130	−0.0397	0.1674	0.021*	
C36	1.0415 (2)	0.09555 (15)	0.19838 (5)	0.0215 (3)	
H36	1.0433	0.0632	0.2242	0.026*	
C37	1.1188 (2)	0.20578 (15)	0.19500 (5)	0.0221 (4)	
H37	1.1727	0.2470	0.2186	0.027*	
C38	1.1168 (2)	0.25401 (14)	0.15806 (5)	0.0190 (3)	
H38	1.1702	0.3281	0.1561	0.023*	
C39	0.7281 (2)	0.50044 (14)	0.51735 (5)	0.0170 (3)	
C40	0.7726 (2)	0.42625 (14)	0.48243 (5)	0.0189 (3)	
H40	0.7965	0.3461	0.4858	0.023*	
C41	0.7792 (2)	0.47129 (14)	0.44642 (5)	0.0164 (3)	
H41	0.7498	0.5511	0.4441	0.020*	
C42	0.8279 (2)	0.40890 (13)	0.40996 (4)	0.0147 (3)	
C43	0.7975 (2)	0.46376 (13)	0.37293 (5)	0.0154 (3)	
H43	0.7472	0.5393	0.3720	0.019*	
C44	0.8410 (2)	0.40780 (14)	0.33775 (4)	0.0154 (3)	
C45	0.9157 (2)	0.29754 (14)	0.33880 (5)	0.0157 (3)	
C46	0.9474 (2)	0.24305 (14)	0.37545 (5)	0.0168 (3)	
H46	0.9995	0.1680	0.3763	0.020*	
C47	0.9029 (2)	0.29816 (14)	0.41062 (5)	0.0168 (3)	
H47	0.9238	0.2602	0.4355	0.020*	
C48	0.7104 (2)	0.49980 (13)	0.59074 (5)	0.0149 (3)	
H48	0.7608	0.5758	0.5924	0.018*	
C49	0.6802 (2)	0.44107 (13)	0.55364 (4)	0.0153 (3)	
C50	0.6039 (2)	0.32813 (14)	0.55080 (5)	0.0175 (3)	
H50	0.5860	0.2869	0.5254	0.021*	
C51	0.5557 (2)	0.27817 (14)	0.58458 (5)	0.0177 (3)	
H51	0.5006	0.2036	0.5822	0.021*	
C52	0.5868 (2)	0.33593 (13)	0.62308 (5)	0.0155 (3)	
C53	0.6675 (2)	0.44834 (13)	0.62628 (5)	0.0146 (3)	
C54	0.7031 (2)	0.50529 (14)	0.66494 (5)	0.0183 (3)	
H54	0.7571	0.5802	0.6674	0.022*	
C55	0.6602 (2)	0.45286 (15)	0.69874 (5)	0.0213 (3)	
H55	0.6856	0.4914	0.7245	0.026*	
C56	0.5787 (2)	0.34214 (15)	0.69557 (5)	0.0227 (4)	
H56	0.5489	0.3068	0.7192	0.027*	
C57	0.5420 (2)	0.28511 (14)	0.65881 (5)	0.0192 (3)	
H57	0.4861	0.2108	0.6571	0.023*	
C58	0.2305 (2)	0.00346 (14)	0.51642 (5)	0.0172 (3)	
C59	0.2745 (2)	0.06893 (14)	0.48147 (5)	0.0188 (3)	
H59	0.2994	0.1498	0.4848	0.023*	
C60	0.2794 (2)	0.01502 (14)	0.44534 (5)	0.0168 (3)	
H60	0.2490	−0.0653	0.4431	0.020*	
C61	0.3273 (2)	0.06839 (13)	0.40872 (4)	0.0154 (3)	
C62	0.2955 (2)	0.00500 (14)	0.37185 (5)	0.0158 (3)	
H62	0.2441	−0.0706	0.3711	0.019*	
C63	0.3385 (2)	0.05196 (14)	0.33638 (4)	0.0158 (3)	
C64	0.4154 (2)	0.16158 (14)	0.33743 (4)	0.0158 (3)	
C65	0.4487 (2)	0.22523 (14)	0.37399 (5)	0.0169 (3)	
H65	0.5018	0.3003	0.3747	0.020*	
C66	0.4045 (2)	0.17903 (13)	0.40931 (5)	0.0166 (3)	
H66	0.4268	0.2229	0.4342	0.020*	
C67	0.2121 (2)	0.02260 (13)	0.58983 (5)	0.0149 (3)	
H67	0.2623	−0.0528	0.5917	0.018*	
C68	0.1818 (2)	0.07219 (13)	0.55265 (4)	0.0155 (3)	
C69	0.1058 (2)	0.18394 (14)	0.54959 (5)	0.0177 (3)	
H69	0.0885	0.2187	0.5241	0.021*	
C70	0.0567 (2)	0.24240 (14)	0.58322 (5)	0.0172 (3)	
H70	0.0012	0.3161	0.5806	0.021*	
C71	0.0880 (2)	0.19411 (13)	0.62188 (5)	0.0154 (3)	
C72	0.1689 (2)	0.08319 (13)	0.62524 (4)	0.0142 (3)	
C73	0.2038 (2)	0.03555 (14)	0.66403 (5)	0.0176 (3)	
H73	0.2579	−0.0386	0.6666	0.021*	
C74	0.1598 (2)	0.09629 (15)	0.69779 (5)	0.0208 (3)	
H74	0.1846	0.0642	0.7236	0.025*	
C75	0.0781 (2)	0.20611 (15)	0.69434 (5)	0.0212 (3)	
H75	0.0481	0.2473	0.7179	0.025*	
C76	0.0416 (2)	0.25385 (14)	0.65738 (5)	0.0185 (3)	
H76	−0.0151	0.3273	0.6554	0.022*	

### Atomic displacement parameters (Å^2^)

**Table:**

	*U*^11^	*U*^22^	*U*^33^	*U*^12^	*U*^13^	*U*^23^
Cl1	0.0263 (2)	0.0234 (2)	0.01441 (18)	−0.00252 (17)	0.00050 (15)	0.00574 (14)
Cl2	0.0233 (2)	0.0231 (2)	0.01752 (18)	−0.00437 (16)	−0.00162 (15)	−0.00247 (14)
Cl3	0.0270 (2)	0.0200 (2)	0.01394 (17)	0.00279 (16)	0.00222 (15)	−0.00196 (14)
Cl4	0.0227 (2)	0.0206 (2)	0.01712 (18)	0.00368 (16)	−0.00198 (15)	0.00331 (14)
Cl5	0.0267 (2)	0.0216 (2)	0.01365 (17)	0.00192 (16)	0.00179 (15)	0.00411 (14)
Cl6	0.0249 (2)	0.0224 (2)	0.01806 (18)	0.00308 (16)	0.00624 (15)	−0.00309 (14)
Cl7	0.0281 (2)	0.0203 (2)	0.01389 (17)	−0.00261 (16)	0.00228 (15)	−0.00151 (14)
Cl8	0.0238 (2)	0.0222 (2)	0.01798 (18)	−0.00305 (16)	0.00538 (15)	0.00522 (14)
O1	0.0347 (7)	0.0173 (6)	0.0191 (6)	−0.0015 (5)	−0.0002 (5)	0.0021 (4)
O2	0.0332 (7)	0.0174 (6)	0.0184 (6)	0.0001 (5)	−0.0009 (5)	0.0001 (4)
O3	0.0353 (7)	0.0173 (6)	0.0183 (6)	0.0016 (5)	0.0048 (5)	0.0015 (4)
O4	0.0342 (7)	0.0177 (6)	0.0183 (6)	−0.0009 (5)	0.0041 (5)	0.0002 (4)
C1	0.0172 (8)	0.0176 (8)	0.0142 (7)	−0.0026 (6)	0.0023 (6)	0.0018 (6)
C2	0.0214 (9)	0.0175 (8)	0.0176 (8)	−0.0031 (7)	0.0007 (6)	0.0010 (6)
C3	0.0161 (8)	0.0168 (8)	0.0165 (7)	−0.0007 (6)	0.0013 (6)	0.0000 (6)
C4	0.0131 (8)	0.0165 (8)	0.0160 (7)	0.0014 (6)	0.0017 (6)	0.0011 (6)
C5	0.0140 (8)	0.0157 (7)	0.0180 (7)	−0.0008 (6)	0.0018 (6)	0.0015 (6)
C6	0.0146 (8)	0.0180 (8)	0.0136 (7)	0.0017 (6)	0.0022 (6)	0.0036 (6)
C7	0.0137 (8)	0.0176 (8)	0.0151 (7)	0.0013 (6)	0.0008 (6)	−0.0015 (6)
C8	0.0170 (8)	0.0142 (7)	0.0198 (7)	−0.0015 (6)	0.0041 (6)	0.0017 (6)
C9	0.0181 (8)	0.0167 (8)	0.0152 (7)	0.0005 (6)	0.0030 (6)	0.0030 (6)
C10	0.0132 (8)	0.0133 (7)	0.0174 (7)	−0.0008 (6)	0.0014 (6)	0.0005 (6)
C11	0.0155 (8)	0.0164 (8)	0.0143 (7)	−0.0026 (6)	0.0018 (6)	0.0027 (6)
C12	0.0189 (9)	0.0188 (8)	0.0167 (7)	−0.0021 (7)	0.0042 (6)	−0.0029 (6)
C13	0.0175 (8)	0.0151 (8)	0.0211 (8)	0.0008 (6)	0.0050 (6)	−0.0002 (6)
C14	0.0110 (8)	0.0150 (7)	0.0194 (7)	−0.0019 (6)	0.0029 (6)	0.0011 (6)
C15	0.0125 (8)	0.0159 (7)	0.0160 (7)	−0.0015 (6)	0.0017 (6)	0.0015 (6)
C16	0.0161 (8)	0.0184 (8)	0.0174 (7)	−0.0002 (6)	0.0016 (6)	−0.0020 (6)
C17	0.0201 (9)	0.0249 (9)	0.0159 (7)	−0.0007 (7)	0.0006 (6)	−0.0002 (6)
C18	0.0189 (9)	0.0251 (9)	0.0196 (8)	−0.0016 (7)	−0.0025 (6)	0.0061 (6)
C19	0.0162 (8)	0.0167 (8)	0.0234 (8)	0.0006 (6)	0.0015 (6)	0.0042 (6)
C20	0.0173 (8)	0.0193 (8)	0.0147 (7)	0.0020 (6)	0.0021 (6)	0.0014 (6)
C21	0.0214 (9)	0.0174 (8)	0.0177 (8)	0.0024 (7)	0.0008 (6)	0.0014 (6)
C22	0.0157 (8)	0.0165 (8)	0.0175 (7)	−0.0002 (6)	0.0015 (6)	0.0030 (6)
C23	0.0136 (8)	0.0162 (7)	0.0150 (7)	−0.0014 (6)	0.0013 (6)	0.0018 (6)
C24	0.0145 (8)	0.0145 (7)	0.0164 (7)	−0.0005 (6)	0.0022 (6)	0.0011 (6)
C25	0.0148 (8)	0.0171 (8)	0.0142 (7)	−0.0027 (6)	0.0030 (6)	−0.0013 (6)
C26	0.0131 (8)	0.0175 (8)	0.0155 (7)	−0.0007 (6)	0.0003 (6)	0.0030 (6)
C27	0.0152 (8)	0.0144 (7)	0.0207 (8)	0.0012 (6)	0.0028 (6)	0.0002 (6)
C28	0.0175 (8)	0.0171 (8)	0.0155 (7)	−0.0003 (6)	0.0022 (6)	−0.0012 (6)
C29	0.0137 (8)	0.0139 (7)	0.0174 (7)	0.0007 (6)	0.0022 (6)	0.0013 (6)
C30	0.0142 (8)	0.0166 (8)	0.0154 (7)	0.0022 (6)	0.0020 (6)	0.0006 (6)
C31	0.0204 (9)	0.0193 (8)	0.0152 (7)	0.0017 (7)	0.0046 (6)	0.0046 (6)
C32	0.0167 (8)	0.0155 (8)	0.0205 (8)	−0.0009 (6)	0.0061 (6)	0.0021 (6)
C33	0.0127 (8)	0.0161 (7)	0.0178 (7)	0.0011 (6)	0.0026 (6)	0.0013 (6)
C34	0.0132 (8)	0.0162 (7)	0.0156 (7)	0.0021 (6)	0.0023 (6)	0.0009 (6)
C35	0.0157 (8)	0.0182 (8)	0.0186 (8)	0.0020 (6)	0.0021 (6)	0.0028 (6)
C36	0.0236 (9)	0.0249 (9)	0.0158 (7)	0.0036 (7)	0.0012 (6)	0.0018 (6)
C37	0.0209 (9)	0.0256 (9)	0.0187 (8)	0.0019 (7)	−0.0013 (6)	−0.0034 (6)
C38	0.0163 (8)	0.0183 (8)	0.0220 (8)	−0.0007 (6)	0.0014 (6)	−0.0021 (6)
C39	0.0186 (8)	0.0180 (8)	0.0142 (7)	0.0018 (6)	0.0000 (6)	0.0017 (6)
C40	0.0210 (9)	0.0182 (8)	0.0179 (8)	0.0035 (7)	0.0033 (6)	0.0015 (6)
C41	0.0156 (8)	0.0165 (8)	0.0169 (7)	0.0001 (6)	0.0020 (6)	−0.0001 (6)
C42	0.0140 (8)	0.0156 (7)	0.0144 (7)	−0.0027 (6)	0.0014 (6)	0.0008 (6)
C43	0.0140 (8)	0.0152 (7)	0.0173 (7)	−0.0003 (6)	0.0018 (6)	0.0022 (6)
C44	0.0144 (8)	0.0178 (8)	0.0140 (7)	−0.0015 (6)	0.0008 (6)	0.0038 (6)
C45	0.0141 (8)	0.0175 (8)	0.0155 (7)	−0.0010 (6)	0.0031 (6)	−0.0024 (6)
C46	0.0155 (8)	0.0147 (7)	0.0200 (8)	0.0004 (6)	0.0006 (6)	0.0012 (6)
C47	0.0180 (8)	0.0172 (8)	0.0150 (7)	−0.0006 (6)	0.0000 (6)	0.0032 (6)
C48	0.0144 (8)	0.0135 (7)	0.0167 (7)	0.0007 (6)	0.0008 (6)	0.0014 (6)
C49	0.0136 (8)	0.0173 (8)	0.0151 (7)	0.0024 (6)	0.0010 (6)	0.0019 (6)
C50	0.0175 (8)	0.0175 (8)	0.0166 (7)	0.0021 (6)	−0.0013 (6)	−0.0023 (6)
C51	0.0142 (8)	0.0152 (8)	0.0230 (8)	−0.0015 (6)	−0.0008 (6)	0.0008 (6)
C52	0.0117 (8)	0.0163 (8)	0.0185 (7)	0.0019 (6)	0.0008 (6)	0.0028 (6)
C53	0.0107 (8)	0.0172 (8)	0.0161 (7)	0.0014 (6)	0.0013 (6)	0.0014 (6)
C54	0.0165 (8)	0.0198 (8)	0.0182 (8)	0.0005 (6)	0.0006 (6)	0.0002 (6)
C55	0.0203 (9)	0.0295 (9)	0.0140 (7)	0.0033 (7)	0.0015 (6)	−0.0007 (6)
C56	0.0220 (9)	0.0278 (9)	0.0195 (8)	0.0033 (7)	0.0050 (7)	0.0087 (7)
C57	0.0158 (8)	0.0185 (8)	0.0241 (8)	0.0009 (6)	0.0032 (6)	0.0069 (6)
C58	0.0179 (8)	0.0193 (8)	0.0139 (7)	−0.0020 (6)	−0.0005 (6)	0.0010 (6)
C59	0.0212 (9)	0.0182 (8)	0.0171 (7)	−0.0031 (7)	0.0024 (6)	0.0017 (6)
C60	0.0162 (8)	0.0174 (8)	0.0170 (7)	−0.0002 (6)	0.0014 (6)	0.0024 (6)
C61	0.0130 (8)	0.0172 (8)	0.0160 (7)	0.0014 (6)	0.0016 (6)	0.0022 (6)
C62	0.0140 (8)	0.0152 (7)	0.0182 (7)	−0.0006 (6)	0.0012 (6)	0.0016 (6)
C63	0.0146 (8)	0.0183 (8)	0.0142 (7)	0.0019 (6)	0.0010 (6)	−0.0011 (6)
C64	0.0141 (8)	0.0188 (8)	0.0151 (7)	0.0013 (6)	0.0031 (6)	0.0054 (6)
C65	0.0159 (8)	0.0145 (7)	0.0200 (8)	−0.0015 (6)	0.0007 (6)	0.0018 (6)
C66	0.0175 (8)	0.0164 (8)	0.0153 (7)	0.0003 (6)	−0.0001 (6)	−0.0006 (6)
C67	0.0141 (8)	0.0139 (7)	0.0167 (7)	−0.0012 (6)	0.0006 (6)	0.0017 (6)
C68	0.0154 (8)	0.0165 (8)	0.0146 (7)	−0.0034 (6)	0.0011 (6)	0.0008 (6)
C69	0.0182 (8)	0.0185 (8)	0.0159 (7)	−0.0021 (6)	−0.0016 (6)	0.0042 (6)
C70	0.0152 (8)	0.0152 (8)	0.0208 (8)	0.0016 (6)	−0.0006 (6)	0.0029 (6)
C71	0.0115 (8)	0.0165 (8)	0.0182 (7)	−0.0020 (6)	0.0005 (6)	0.0018 (6)
C72	0.0109 (8)	0.0160 (7)	0.0157 (7)	−0.0022 (6)	0.0007 (6)	0.0021 (6)
C73	0.0170 (8)	0.0183 (8)	0.0173 (7)	−0.0001 (6)	0.0007 (6)	0.0030 (6)
C74	0.0240 (9)	0.0232 (8)	0.0150 (7)	−0.0023 (7)	0.0012 (6)	0.0025 (6)
C75	0.0207 (9)	0.0238 (9)	0.0194 (8)	−0.0020 (7)	0.0052 (6)	−0.0036 (6)
C76	0.0161 (8)	0.0165 (8)	0.0226 (8)	0.0003 (6)	0.0024 (6)	−0.0013 (6)

### Geometric parameters (Å, °)

**Table:**

Cl1—C6	1.7246 (15)	C35—H35	0.9500
Cl2—C7	1.7275 (16)	C36—C37	1.409 (2)
Cl3—C25	1.7244 (15)	C36—H36	0.9500
Cl4—C26	1.7258 (15)	C37—C38	1.369 (2)
Cl5—C44	1.7239 (15)	C37—H37	0.9500
Cl6—C45	1.7255 (16)	C38—H38	0.9500
Cl7—C63	1.7229 (16)	C39—C40	1.484 (2)
Cl8—C64	1.7286 (15)	C39—C49	1.491 (2)
O1—C1	1.2212 (19)	C40—C41	1.332 (2)
O2—C20	1.2209 (19)	C40—H40	0.9500
O3—C39	1.2241 (19)	C41—C42	1.468 (2)
O4—C58	1.2193 (19)	C41—H41	0.9500
C1—C2	1.482 (2)	C42—C47	1.396 (2)
C1—C11	1.495 (2)	C42—C43	1.405 (2)
C2—C3	1.337 (2)	C43—C44	1.386 (2)
C2—H2	0.9500	C43—H43	0.9500
C3—C4	1.467 (2)	C44—C45	1.390 (2)
C3—H3	0.9500	C45—C46	1.392 (2)
C4—C9	1.397 (2)	C46—C47	1.384 (2)
C4—C5	1.400 (2)	C46—H46	0.9500
C5—C6	1.387 (2)	C47—H47	0.9500
C5—H5	0.9500	C48—C49	1.380 (2)
C6—C7	1.389 (2)	C48—C53	1.414 (2)
C7—C8	1.391 (2)	C48—H48	0.9500
C8—C9	1.384 (2)	C49—C50	1.417 (2)
C8—H8	0.9500	C50—C51	1.368 (2)
C9—H9	0.9500	C50—H50	0.9500
C10—C11	1.379 (2)	C51—C52	1.415 (2)
C10—C15	1.414 (2)	C51—H51	0.9500
C10—H10	0.9500	C52—C57	1.421 (2)
C11—C12	1.417 (2)	C52—C53	1.424 (2)
C12—C13	1.370 (2)	C53—C54	1.419 (2)
C12—H12	0.9500	C54—C55	1.369 (2)
C13—C14	1.418 (2)	C54—H54	0.9500
C13—H13	0.9500	C55—C56	1.409 (2)
C14—C19	1.420 (2)	C55—H55	0.9500
C14—C15	1.424 (2)	C56—C57	1.365 (2)
C15—C16	1.420 (2)	C56—H56	0.9500
C16—C17	1.368 (2)	C57—H57	0.9500
C16—H16	0.9500	C58—C59	1.480 (2)
C17—C18	1.411 (2)	C58—C68	1.497 (2)
C17—H17	0.9500	C59—C60	1.336 (2)
C18—C19	1.366 (2)	C59—H59	0.9500
C18—H18	0.9500	C60—C61	1.468 (2)
C19—H19	0.9500	C60—H60	0.9500
C20—C21	1.482 (2)	C61—C66	1.398 (2)
C20—C30	1.492 (2)	C61—C62	1.399 (2)
C21—C22	1.335 (2)	C62—C63	1.389 (2)
C21—H21	0.9500	C62—H62	0.9500
C22—C23	1.468 (2)	C63—C64	1.386 (2)
C22—H22	0.9500	C64—C65	1.393 (2)
C23—C28	1.400 (2)	C65—C66	1.384 (2)
C23—C24	1.400 (2)	C65—H65	0.9500
C24—C25	1.390 (2)	C66—H66	0.9500
C24—H24	0.9500	C67—C68	1.381 (2)
C25—C26	1.386 (2)	C67—C72	1.415 (2)
C26—C27	1.395 (2)	C67—H67	0.9500
C27—C28	1.384 (2)	C68—C69	1.413 (2)
C27—H27	0.9500	C69—C70	1.370 (2)
C28—H28	0.9500	C69—H69	0.9500
C29—C30	1.383 (2)	C70—C71	1.419 (2)
C29—C34	1.413 (2)	C70—H70	0.9500
C29—H29	0.9500	C71—C72	1.420 (2)
C30—C31	1.417 (2)	C71—C76	1.421 (2)
C31—C32	1.368 (2)	C72—C73	1.422 (2)
C31—H31	0.9500	C73—C74	1.373 (2)
C32—C33	1.420 (2)	C73—H73	0.9500
C32—H32	0.9500	C74—C75	1.411 (2)
C33—C38	1.418 (2)	C74—H74	0.9500
C33—C34	1.422 (2)	C75—C76	1.368 (2)
C34—C35	1.421 (2)	C75—H75	0.9500
C35—C36	1.369 (2)	C76—H76	0.9500
			
O1—C1—C2	121.90 (14)	O3—C39—C40	121.64 (14)
O1—C1—C11	120.52 (14)	O3—C39—C49	120.78 (14)
C2—C1—C11	117.57 (13)	C40—C39—C49	117.58 (13)
C3—C2—C1	120.43 (15)	C41—C40—C39	120.45 (15)
C3—C2—H2	119.8	C41—C40—H40	119.8
C1—C2—H2	119.8	C39—C40—H40	119.8
C2—C3—C4	125.77 (15)	C40—C41—C42	125.94 (15)
C2—C3—H3	117.1	C40—C41—H41	117.0
C4—C3—H3	117.1	C42—C41—H41	117.0
C9—C4—C5	118.88 (14)	C47—C42—C43	118.94 (14)
C9—C4—C3	122.70 (14)	C47—C42—C41	122.89 (13)
C5—C4—C3	118.42 (14)	C43—C42—C41	118.16 (14)
C6—C5—C4	120.36 (14)	C44—C43—C42	120.05 (14)
C6—C5—H5	119.8	C44—C43—H43	120.0
C4—C5—H5	119.8	C42—C43—H43	120.0
C5—C6—C7	120.10 (14)	C43—C44—C45	120.46 (13)
C5—C6—Cl1	119.34 (12)	C43—C44—Cl5	119.29 (12)
C7—C6—Cl1	120.56 (12)	C45—C44—Cl5	120.24 (12)
C6—C7—C8	120.04 (14)	C44—C45—C46	119.78 (14)
C6—C7—Cl2	120.74 (12)	C44—C45—Cl6	120.85 (12)
C8—C7—Cl2	119.21 (12)	C46—C45—Cl6	119.37 (12)
C9—C8—C7	119.89 (15)	C47—C46—C45	119.99 (15)
C9—C8—H8	120.1	C47—C46—H46	120.0
C7—C8—H8	120.1	C45—C46—H46	120.0
C8—C9—C4	120.72 (14)	C46—C47—C42	120.77 (14)
C8—C9—H9	119.6	C46—C47—H47	119.6
C4—C9—H9	119.6	C42—C47—H47	119.6
C11—C10—C15	120.81 (14)	C49—C48—C53	120.84 (14)
C11—C10—H10	119.6	C49—C48—H48	119.6
C15—C10—H10	119.6	C53—C48—H48	119.6
C10—C11—C12	119.97 (14)	C48—C49—C50	119.95 (14)
C10—C11—C1	118.11 (14)	C48—C49—C39	118.11 (14)
C12—C11—C1	121.91 (14)	C50—C49—C39	121.93 (14)
C13—C12—C11	120.29 (14)	C51—C50—C49	120.23 (14)
C13—C12—H12	119.9	C51—C50—H50	119.9
C11—C12—H12	119.9	C49—C50—H50	119.9
C12—C13—C14	120.88 (15)	C50—C51—C52	121.00 (15)
C12—C13—H13	119.6	C50—C51—H51	119.5
C14—C13—H13	119.6	C52—C51—H51	119.5
C13—C14—C19	122.14 (14)	C51—C52—C57	122.25 (15)
C13—C14—C15	118.93 (14)	C51—C52—C53	119.02 (14)
C19—C14—C15	118.93 (14)	C57—C52—C53	118.72 (14)
C10—C15—C16	121.85 (14)	C48—C53—C54	121.89 (14)
C10—C15—C14	119.05 (14)	C48—C53—C52	118.90 (14)
C16—C15—C14	119.10 (14)	C54—C53—C52	119.21 (14)
C17—C16—C15	120.36 (15)	C55—C54—C53	120.37 (15)
C17—C16—H16	119.8	C55—C54—H54	119.8
C15—C16—H16	119.8	C53—C54—H54	119.8
C16—C17—C18	120.52 (15)	C54—C55—C56	120.47 (15)
C16—C17—H17	119.7	C54—C55—H55	119.8
C18—C17—H17	119.7	C56—C55—H55	119.8
C19—C18—C17	120.64 (14)	C57—C56—C55	120.64 (15)
C19—C18—H18	119.7	C57—C56—H56	119.7
C17—C18—H18	119.7	C55—C56—H56	119.7
C18—C19—C14	120.44 (15)	C56—C57—C52	120.57 (15)
C18—C19—H19	119.8	C56—C57—H57	119.7
C14—C19—H19	119.8	C52—C57—H57	119.7
O2—C20—C21	121.80 (14)	O4—C58—C59	121.78 (15)
O2—C20—C30	120.90 (14)	O4—C58—C68	120.78 (14)
C21—C20—C30	117.30 (14)	C59—C58—C68	117.44 (14)
C22—C21—C20	120.46 (15)	C60—C59—C58	120.44 (15)
C22—C21—H21	119.8	C60—C59—H59	119.8
C20—C21—H21	119.8	C58—C59—H59	119.8
C21—C22—C23	125.74 (15)	C59—C60—C61	126.00 (15)
C21—C22—H22	117.1	C59—C60—H60	117.0
C23—C22—H22	117.1	C61—C60—H60	117.0
C28—C23—C24	118.84 (13)	C66—C61—C62	118.95 (14)
C28—C23—C22	122.59 (14)	C66—C61—C60	122.73 (14)
C24—C23—C22	118.57 (14)	C62—C61—C60	118.31 (14)
C25—C24—C23	120.42 (14)	C63—C62—C61	120.37 (14)
C25—C24—H24	119.8	C63—C62—H62	119.8
C23—C24—H24	119.8	C61—C62—H62	119.8
C26—C25—C24	120.08 (14)	C64—C63—C62	120.05 (14)
C26—C25—Cl3	120.54 (12)	C64—C63—Cl7	120.53 (12)
C24—C25—Cl3	119.38 (12)	C62—C63—Cl7	119.41 (12)
C25—C26—C27	120.03 (14)	C63—C64—C65	120.10 (14)
C25—C26—Cl4	120.98 (12)	C63—C64—Cl8	120.92 (12)
C27—C26—Cl4	118.99 (12)	C65—C64—Cl8	118.98 (12)
C28—C27—C26	119.96 (14)	C66—C65—C64	119.95 (15)
C28—C27—H27	120.0	C66—C65—H65	120.0
C26—C27—H27	120.0	C64—C65—H65	120.0
C27—C28—C23	120.66 (14)	C65—C66—C61	120.57 (14)
C27—C28—H28	119.7	C65—C66—H66	119.7
C23—C28—H28	119.7	C61—C66—H66	119.7
C30—C29—C34	120.75 (14)	C68—C67—C72	120.57 (14)
C30—C29—H29	119.6	C68—C67—H67	119.7
C34—C29—H29	119.6	C72—C67—H67	119.7
C29—C30—C31	119.72 (14)	C67—C68—C69	120.09 (14)
C29—C30—C20	118.11 (14)	C67—C68—C58	117.86 (14)
C31—C30—C20	122.17 (13)	C69—C68—C58	122.05 (13)
C32—C31—C30	120.61 (14)	C70—C69—C68	120.37 (14)
C32—C31—H31	119.7	C70—C69—H69	119.8
C30—C31—H31	119.7	C68—C69—H69	119.8
C31—C32—C33	120.68 (14)	C69—C70—C71	120.73 (15)
C31—C32—H32	119.7	C69—C70—H70	119.6
C33—C32—H32	119.7	C71—C70—H70	119.6
C38—C33—C32	121.89 (15)	C70—C71—C72	119.03 (14)
C38—C33—C34	119.14 (14)	C70—C71—C76	121.91 (14)
C32—C33—C34	118.96 (14)	C72—C71—C76	119.06 (14)
C29—C34—C35	121.81 (14)	C67—C72—C71	119.14 (13)
C29—C34—C33	119.22 (14)	C67—C72—C73	121.65 (14)
C35—C34—C33	118.97 (14)	C71—C72—C73	119.21 (14)
C36—C35—C34	120.39 (15)	C74—C73—C72	120.26 (15)
C36—C35—H35	119.8	C74—C73—H73	119.9
C34—C35—H35	119.8	C72—C73—H73	119.9
C35—C36—C37	120.54 (15)	C73—C74—C75	120.41 (15)
C35—C36—H36	119.7	C73—C74—H74	119.8
C37—C36—H36	119.7	C75—C74—H74	119.8
C38—C37—C36	120.57 (15)	C76—C75—C74	120.67 (15)
C38—C37—H37	119.7	C76—C75—H75	119.7
C36—C37—H37	119.7	C74—C75—H75	119.7
C37—C38—C33	120.38 (15)	C75—C76—C71	120.37 (15)
C37—C38—H38	119.8	C75—C76—H76	119.8
C33—C38—H38	119.8	C71—C76—H76	119.8
			
O1—C1—C2—C3	16.4 (3)	O3—C39—C40—C41	−14.9 (3)
C11—C1—C2—C3	−164.66 (15)	C49—C39—C40—C41	165.71 (15)
C1—C2—C3—C4	−177.23 (15)	C39—C40—C41—C42	177.56 (15)
C2—C3—C4—C9	11.3 (3)	C40—C41—C42—C47	−11.3 (3)
C2—C3—C4—C5	−168.55 (16)	C40—C41—C42—C43	168.90 (16)
C9—C4—C5—C6	−0.1 (2)	C47—C42—C43—C44	0.4 (2)
C3—C4—C5—C6	179.73 (14)	C41—C42—C43—C44	−179.80 (14)
C4—C5—C6—C7	0.3 (2)	C42—C43—C44—C45	−0.3 (2)
C4—C5—C6—Cl1	−179.48 (12)	C42—C43—C44—Cl5	−179.52 (12)
C5—C6—C7—C8	0.2 (2)	C43—C44—C45—C46	−0.1 (2)
Cl1—C6—C7—C8	179.99 (12)	Cl5—C44—C45—C46	179.02 (12)
C5—C6—C7—Cl2	178.97 (12)	C43—C44—C45—Cl6	−179.46 (12)
Cl1—C6—C7—Cl2	−1.26 (19)	Cl5—C44—C45—Cl6	−0.3 (2)
C6—C7—C8—C9	−0.9 (2)	C44—C45—C46—C47	0.6 (2)
Cl2—C7—C8—C9	−179.68 (12)	Cl6—C45—C46—C47	179.97 (12)
C7—C8—C9—C4	1.1 (2)	C45—C46—C47—C42	−0.6 (2)
C5—C4—C9—C8	−0.6 (2)	C43—C42—C47—C46	0.1 (2)
C3—C4—C9—C8	179.58 (15)	C41—C42—C47—C46	−179.70 (15)
C15—C10—C11—C12	−0.5 (2)	C53—C48—C49—C50	0.5 (2)
C15—C10—C11—C1	−179.89 (14)	C53—C48—C49—C39	179.83 (14)
O1—C1—C11—C10	25.3 (2)	O3—C39—C49—C48	−25.8 (2)
C2—C1—C11—C10	−153.71 (15)	C40—C39—C49—C48	153.58 (15)
O1—C1—C11—C12	−154.15 (16)	O3—C39—C49—C50	153.49 (16)
C2—C1—C11—C12	26.9 (2)	C40—C39—C49—C50	−27.1 (2)
C10—C11—C12—C13	−2.0 (2)	C48—C49—C50—C51	1.7 (2)
C1—C11—C12—C13	177.42 (15)	C39—C49—C50—C51	−177.61 (15)
C11—C12—C13—C14	2.7 (2)	C49—C50—C51—C52	−2.3 (2)
C12—C13—C14—C19	178.56 (15)	C50—C51—C52—C57	−178.52 (15)
C12—C13—C14—C15	−0.9 (2)	C50—C51—C52—C53	0.8 (2)
C11—C10—C15—C16	−177.72 (15)	C49—C48—C53—C54	177.73 (14)
C11—C10—C15—C14	2.2 (2)	C49—C48—C53—C52	−2.0 (2)
C13—C14—C15—C10	−1.5 (2)	C51—C52—C53—C48	1.4 (2)
C19—C14—C15—C10	179.02 (14)	C57—C52—C53—C48	−179.30 (14)
C13—C14—C15—C16	178.41 (14)	C51—C52—C53—C54	−178.39 (14)
C19—C14—C15—C16	−1.1 (2)	C57—C52—C53—C54	0.9 (2)
C10—C15—C16—C17	−179.55 (15)	C48—C53—C54—C55	−179.85 (15)
C14—C15—C16—C17	0.6 (2)	C52—C53—C54—C55	−0.1 (2)
C15—C16—C17—C18	0.0 (3)	C53—C54—C55—C56	−0.6 (2)
C16—C17—C18—C19	0.0 (3)	C54—C55—C56—C57	0.4 (3)
C17—C18—C19—C14	−0.6 (3)	C55—C56—C57—C52	0.5 (3)
C13—C14—C19—C18	−178.40 (15)	C51—C52—C57—C56	178.14 (15)
C15—C14—C19—C18	1.1 (2)	C53—C52—C57—C56	−1.2 (2)
O2—C20—C21—C22	−15.5 (3)	O4—C58—C59—C60	15.8 (3)
C30—C20—C21—C22	165.11 (15)	C68—C58—C59—C60	−164.81 (15)
C20—C21—C22—C23	177.45 (15)	C58—C59—C60—C61	−177.62 (15)
C21—C22—C23—C28	−10.3 (3)	C59—C60—C61—C66	11.6 (3)
C21—C22—C23—C24	169.81 (16)	C59—C60—C61—C62	−168.95 (16)
C28—C23—C24—C25	0.3 (2)	C66—C61—C62—C63	−0.6 (2)
C22—C23—C24—C25	−179.82 (14)	C60—C61—C62—C63	179.96 (14)
C23—C24—C25—C26	0.1 (2)	C61—C62—C63—C64	0.8 (2)
C23—C24—C25—Cl3	179.94 (12)	C61—C62—C63—Cl7	179.76 (12)
C24—C25—C26—C27	−0.7 (2)	C62—C63—C64—C65	−0.4 (2)
Cl3—C25—C26—C27	179.46 (12)	Cl7—C63—C64—C65	−179.36 (12)
C24—C25—C26—Cl4	−179.92 (12)	C62—C63—C64—Cl8	178.92 (12)
Cl3—C25—C26—Cl4	0.3 (2)	Cl7—C63—C64—Cl8	−0.02 (19)
C25—C26—C27—C28	0.9 (2)	C63—C64—C65—C66	−0.2 (2)
Cl4—C26—C27—C28	−179.85 (12)	Cl8—C64—C65—C66	−179.51 (12)
C26—C27—C28—C23	−0.5 (2)	C64—C65—C66—C61	0.4 (2)
C24—C23—C28—C27	0.0 (2)	C62—C61—C66—C65	0.0 (2)
C22—C23—C28—C27	−179.97 (15)	C60—C61—C66—C65	179.44 (15)
C34—C29—C30—C31	0.2 (2)	C72—C67—C68—C69	−0.5 (2)
C34—C29—C30—C20	179.82 (14)	C72—C67—C68—C58	179.99 (14)
O2—C20—C30—C29	−25.9 (2)	O4—C58—C68—C67	25.6 (2)
C21—C20—C30—C29	153.50 (15)	C59—C58—C68—C67	−153.76 (15)
O2—C20—C30—C31	153.74 (16)	O4—C58—C68—C69	−153.83 (16)
C21—C20—C30—C31	−26.9 (2)	C59—C58—C68—C69	26.8 (2)
C29—C30—C31—C32	2.0 (2)	C67—C68—C69—C70	−1.9 (2)
C20—C30—C31—C32	−177.58 (15)	C58—C68—C69—C70	177.53 (15)
C30—C31—C32—C33	−2.7 (2)	C68—C69—C70—C71	2.6 (2)
C31—C32—C33—C38	−178.73 (15)	C69—C70—C71—C72	−0.9 (2)
C31—C32—C33—C34	1.2 (2)	C69—C70—C71—C76	178.78 (15)
C30—C29—C34—C35	177.97 (15)	C68—C67—C72—C71	2.3 (2)
C30—C29—C34—C33	−1.7 (2)	C68—C67—C72—C73	−178.02 (15)
C38—C33—C34—C29	−179.09 (14)	C70—C71—C72—C67	−1.6 (2)
C32—C33—C34—C29	1.0 (2)	C76—C71—C72—C67	178.80 (14)
C38—C33—C34—C35	1.3 (2)	C70—C71—C72—C73	178.71 (14)
C32—C33—C34—C35	−178.64 (14)	C76—C71—C72—C73	−0.9 (2)
C29—C34—C35—C36	179.91 (15)	C67—C72—C73—C74	−179.76 (15)
C33—C34—C35—C36	−0.4 (2)	C71—C72—C73—C74	0.0 (2)
C34—C35—C36—C37	−0.3 (3)	C72—C73—C74—C75	0.6 (3)
C35—C36—C37—C38	0.3 (3)	C73—C74—C75—C76	−0.1 (3)
C36—C37—C38—C33	0.5 (3)	C74—C75—C76—C71	−0.9 (3)
C32—C33—C38—C37	178.59 (16)	C70—C71—C76—C75	−178.21 (15)
C34—C33—C38—C37	−1.3 (2)	C72—C71—C76—C75	1.4 (2)
